# New York Academy of Medicine

**Published:** 1860-11

**Authors:** 


### SELECTIONS.

### NEW YORK ACADEMY OF MEDICINE.

*Stated Meeting, Oct. 17th,* 1860.—*Discussion on Tetanus.*—The President, Dr. John Watson, read a paper on tetanus, of which the following is an abstract:—Tetanus may originate as a primary or secondary disease, the former being the idiopathic, the latter the traumatic form. Idiopathic tetanus is rarely seen in this city, though it is not uncommon in certain localities. Traumatic tetanus is not always due to mechanical injuries, but may result from burns, ulceration from frost-bitten limbs, chemical irritants, congenital syphilis. Trismus nascentium, so frequent in warm climates, he has never seen. Tetanus, then, as seen in this city, is assignable to some pre-existing local irritation, which affects innervation of the excito-motory apparatus. The severity of the attack bears no relation to that of the wound, a slight scratch, or abrasion of the skin, having in his observation induced a fatal attack. Tetanic symptoms may supervene immediately after the injury, or be delayed for weeks, even until the wound is healed. An angry wound, however, or an ulcer in which suppuration has been arrested, is more liable to be the precursor of an attack. When tetanus is about to occur, no remedies addressed to the wound, such as removal of the cause, amputation of the limb, &c., can arrest it. Tetanus belongs to the class of self-limiting diseases; it rarely lasts beyond the fifth week, and, when general, seldom subsides before the close of the third week. When about to terminate in health its order of retrocession is rarely the same as its onset, nor is its apparent subsidence always permanent. The author entered minutely into the symptomatology of the disease. In fatal cases death is apt to occur within four or five days, either by asphyxia, spasm of the heart, or exhaustion. Where death occurs during a paroxysm it is more often due to the former cause. Several illustrative cases were adduced by the author, in one of which he performed tracheotomy with only temporary relief. Spasm of the heart as a cause of death in tetanus is denied by some writers, but Dr. Watson reports a case in which the fatal result was attributable to that cause, the muscular fibres of the organ being found hard and rigid like cartilage. A case was also given, fatal after amputation of the arm for its relief. In regard to the mortality from this disease, Dr. W. believes that excluding cases in which the employment of powerful remedies has been excessive, and those cases fatal from the severity of the original injury, not less than one-third, or perhaps one-half of those judiciously treated, recover. Of thirty-three cases, of which he has memoranda, occurring in private and hospital practice, and with every grade of injury, there were eleven recoveries. These successful cases were reported at length, and illustrate the author's mode of treatment, which is assafœtida, wine, and fluid nourishment, or, in other words, support, and guarding against spasm. The assafœtida is administered by the rectum, or in a watery emulsion.

Dr. A. H. Stevens, in reference to the relation of cold as an exciting cause of tetanus, referred to a case of fissure in anno which he had operated upon several years ago. The gentleman lived in the country, and the operation was performed on Sunday, with the understanding that the patient should remain in town until the following Tuesday. Contrary to directions, however, he started for home on the afternoon of the same day, and being exposed during the night to a draught of air in his state-room in the steamboat, caught cold, which in the course of a few days eventuated in tetanus. The treatment consisted of opium, mild enemata, and the free use of beef tea. The case was successful in its issue. Dr. S. further remarked, that he looked upon a person who was suffering from tetanic paroxysms in the same light as one who was being subjected to hard labor; hence the attendant copious perspiration, and consequent exhaustion. He did not think that the necessity for a mild course could be too strongly urged. In conclusion, he expressed his entire concurrence with the views set forth in the paper.

Dr. J. Marion Sims remarked that the late Dr. Drake established the fact in regard to the traumatic tetanus and hydrophobia, occurring in the valley of the Mississippi, that they bore an inverse ratio to each other; that as you go south attacks of the former become more frequent, while the tendency to hydrophobia decreases. So far as the progress of the disease was concerned, all the cases that Dr. Sims had seen were self-limited in character, and also self-curative. He further stated that according to Curling the cases terminated hebdomadally or at the multiple of an hebdomadal period, and that if the disease lasted over a week there was a probability of recovery. Dr. S. advocated the sustaining treatment, but stated that he had seen but very few recoveries take place in the south, where he believed the disease was more fatal in its tendency than elsewhere. He referred to a case which occurred to him fifteen years ago at the south, of a negro who was seized with tetanus in consequence of a punctured wound in the foot from a nail. Having on consultation of authorities seen division of the nerve recommended, he determined to perform such an operation upon the post-tibial. This being done, marked amelioration of the symptoms followed, and the patient finally recovered. In regard to *Trismus Nascentium*, he stated that he had published a paper upon that subject, in the *American Jour. Med. Sciences*, some time in 1848, illustrative of his peculiar views of that disease; that he considered it a disease of centric origin by mechanical compression of the medulla oblongata. The first few cases were of such a character as to induce him to believe that the pressure was occasioned by the occipital bone; subsequent observations, however, established the fact that lateral pressure might produce it equally as well. At that time also he advanced some theoretical views as based upon his first few cases, which, however, he was since compelled to retract. He stated that in parturition the occipital bone was depressed and overlapped by the two parietals at the lambdoidal suture, for the purpose of accommodating the diameters of the head to those of the pelvis; and that if the bone was allowed to retain its position there was always danger. It is a disease that very seldom occurs after the ninth or tenth day, but is most usual during the first three or four days of existence. Contrary to the generally-accepted opinion, it is liable to occur in the coldest climates. In conclusion he referred to the following case, occurring in the practice of Dr. Griscom. The child had suffered from the following symptoms during thirty-six hours: borborygma; greenish passages from the bowels; constant sleeplessness; inability to suck; moaning and slight spasmodic twitches. On examination of the cranium, the peculiar abnormal relation already referred to between the occipital and parietal bones was noticed; the child was placed upon its side in such a way that the weight of the head rested along the edge of the os frontis, and in about a minute after the child became perfectly quiet, and slept four hours. An hour after waking all the unpleasant symptoms had subsided, and the little one was able to take the breast.

Dr. McNulty remarked that it was a scientific maxim that, like causes under like circumstances must of necessity produce like results. This being the fact, if this rule was applied to tetanus, it would be found that the disease was not the result of local injury, inasmuch as very few of the vast number who received such injuries suffered from any tetanic spasms. He thought that it was necessary to suppose in those cases where persons did suffer from the disease, that a tetanic diathesis existed, and that the wound was merely the exciting cause.

Dr. Watson remarked that Dr. McNulty took very singular views of the subject. According to such a theory it would be as well to suppose that hydrophobia did not depend upon the bite of a dog, because every one bitten did not suffer from the disease.

Dr. J. P. Batchelder stated that in every case of tetanus that had come under his observation, the *first* symptom which showed itself was a *stiffness of the muscles of the leg.* He thought that such would be found to be the fact in all cases, if the patients were interrogated with reference to that point. In his experience, if the patient survived the first week with a pulse not over 100, he would get well.

Dr. H. D. Bulkley recollected being told by Dr. Knight of a case of idiopathic tetanus.

Dr. M. G. Porter referred to a case of idiopathic tetanus which occurred to him four or five years ago. The patient was ten years of age; the attack was a very severe one, and recovery was the result of a free use of brandy and beef-tea. From the absence of evidence to the contrary, he considered the case as unquestionably idiopathic in character.

Dr. J. Foster related the history of a case of tetanus which seemed to have been caused on two successive occasions by the administration of bi-chloride of mercury.—*Amer. Med. Times.*

